# Characterization of the Mos10 (Vps60) ESCRT-III complex

**DOI:** 10.1042/BSR20260272

**Published:** 2026-10-05

**Authors:** Joël Vollmar, Thomas Brune, Ralf Kölling

**Affiliations:** Institut für Lebensmittelwissenschaft und Biotechnologie, Fg. Hefegenetik und Gärungstechnologie, Universität Hohenheim, Germany

**Keywords:** endosomal sorting, endosome, lipids, vesicular trafficking, yeast

## Abstract

The endosomal sorting complex required for transport (ESCRT)-III protein Mos10 (also known as Vps60) forms an alternative ESCRT-III complex in yeast. Here, we explored the relationship between the Mos10 complex and the classical Snf7-based complex. Both proteins could in principle form completely independent complexes or mixed complexes. To address the present issue, we examined the composition of ESCRT-III in different mutants of the endosomal protein sorting pathway by co-immunoprecipitation. We found that the formation of the Mos10 complex was dependent on the activity of the AAA+ ATPase Vps4. At the same time, loss of Vps4 activity led to an accumulation of the canonical ESCRT-III proteins Snf7, Vps2, and Vps24. In none of the mutants tested could a complex be detected that contains only Mos10 and no Snf7. We therefore favor the view that Snf7 and Mos10 form mixed complexes, which could play a role in the disassembly of ESCRT-III. To further dissect the interactions among the ESCRT-III subunits, Mos10 mutants were isolated. The L49P mutation in helix α1 altered Mos10 localization, while the L150P mutation in helix α4 abolished membrane association. A potential explanation for the effect of the L49P mutation on Mos10 function is suggested by AlphaFold modeling.

## Introduction

The endosomal sorting complex required for transport (ESCRT)-III complex is involved in a large number of membrane remodeling events in eukaryotic cells. It is involved in the formation and abscission of intraluminal vesicles (ILVs) at multivesicular endosomes (MVBs), in HIV budding, in the abscission step during cell division, in the reformation of the nuclear envelope at the end of mitosis, in membrane repair, in autophagy, and in neuron pruning [1–3]. The ESCRT-III proteins were initially identified in yeast as proteins involved in endosomal protein sorting [4,5]. ESCRT-III is part of a cascade of protein complexes leading to the incorporation of ubiquitinated endocytic cargo proteins into ILVs, bound to be degraded in the vacuole [6–8]. The ESCRT-III protein family consists of eight proteins in yeast and twelve proteins in metazoan cells. Only four of these proteins (Snf7, Vps2, Vps20, and Vps24) are considered to constitute the ESCRT-III complex proper [7]. The other ESCRT-III proteins are called ‘ESCRT-III associated proteins’ [9–11]. Newer results, however, cast doubt on this distinction, since all of the ESCRT-III proteins can assemble into common ESCRT-III complexes [12,13].

We have been especially interested in the function of the Mos10 protein (also known as Vps60). Contrary to other reports [9,11], we observed a strong defect in the sorting of endocytic cargo proteins to the yeast vacuole in *MOS10* deletion mutants [5]. Newer results confirm our findings [13,14]. Initially, it was thought that the ‘ESCRT-III-associated proteins’ Did2 and Mos10 simply act as regulators of Vps4 activity [10,15], but it is now clear that both proteins can assemble into filaments just like the other ESCRT-III proteins [13]. Filament formation appears to be a general property of all ESCRT-III proteins. Mos10 forms an ESCRT-III complex with characteristics similar to the well-studied Snf7-containing complex, but it is still unclear how the two complexes relate to each other [13,16].

At present, several scenarios can be envisioned. The complexes could perform parallel or even completely different functions, or they could act sequentially in the same process. From *in vitro* studies, it has been concluded that Mos10 and Snf7 form completely separate complexes that do not intermix [13]. This is supported by *in vivo* findings that CHMP5/Mos10 complexes and CHMP4B/Snf7 complexes occupy different membrane areas on mammalian lysosomes damaged by the addition of the L-leucyl-L-leucine methyl ester [13]. There are indications that the complexes could act sequentially. The assembly kinetics of the Mos10/CHMP5 complex is slower than the assembly kinetics of the Snf7/CHMP4B complex *in vitro* as well as *in vivo* [13].

But there are also a number of findings that contradict the notion that the two complexes are completely independent of each other. First of all, the two complexes are involved in the same process, which is formation and/or abscission of ILVs at MVBs [17]. What is also difficult to reconcile with a completely separate function of Mos10 and Snf7 is the finding that the formation of the Mos10 complex is dependent on Snf7 [12,16]. Apparently, the presence or activity of Snf7 prepares the endosomal membrane for Mos10 binding. ESCRT-III could be involved in lipid remodeling at the endosomal membrane. We recently found that ESCRT-III interacts with the yeast scramblase homologue Tms1 [18]. Scramblases abolish lipid asymmetry between the two membrane leaflets. An Snf7-induced change in membrane composition at the site of ILV formation could thus be a prerequisite for the formation of the Mos10 complex.

But it is also possible that Mos10 and Snf7 can occur, at least transiently, in the same complex. In an experiment using stable isotope labeling of amino acids in cell culture and mass spectrometry, we pulled out all pertinent ESCRT-III proteins including Snf7 using Mos10-6His as bait [16]. Thus, mixed complexes may exist.

To explore the relationship between Snf7 and Mos10-containing complexes, we now looked at the composition of ESCRT-III in different mutants blocked at different points in the endosomal protein sorting pathway and generated random Mos10 mutants. We found that the recovery of Mos10-containing complexes was dependent on the activity of the AAA+ ATPase Vps4. Based on our data, we favor the view that a mixed complex containing Snf7 and Mos10 is formed at some point in the functional cycle of ESCRT-III. This complex could play a role in the disassembly of ESCRT-III.

## Materials and methods

### Yeast strains and media

For co-immunoprecipitation experiments, yeast cells were grown overnight to exponential phase in YPD medium (1% yeast extract, 2% peptone, and 2% glucose); for fluorescence microscopy, cells were grown in SD/CAS medium (0.67% yeast nitrogen base, 1% casamino acids, 2% glucose, 50 mg/l uracil, and tryptophan) to avoid autofluorescence from the YPD medium. For plasmid selection, tryptophan or uracil was omitted from the medium. The yeast strains used are listed in Supplementary Table S1. The yeast strains are derived from JD52 (J. Dohmen, Cologne, Germany) by the integration of PCR-cassettes into the yeast genome [19]. Plasmids used are listed in Supplementary Table S2.

### Co-immunoprecipitation

Yeast cells were grown overnight in YPD at 30°C to the exponential phase (OD_600_ <1). The optical density of the culture was measured in a photometer at 600 nm. A reading of 1 OD_600_ corresponds to 4 × 10^7^ cells/ml. Fifty OD_600_ units (culture volume × OD_600_) were harvested and washed with cold phosphate-buffered saline (PBS). The cells were resuspended in 200 μl of PBS + protease inhibitors and lysed by shaking with glass beads for 5 min at 4°C. A total of 700 μl of PBS + protease inhibitors and dithiobis (succinimidyl propionate) (Thermo Scientific, Rockford, IL, U.S.A.) to a final concentration of 2 mM were added for crosslinking and incubated at 4°C for 30 min. Crosslinking was quenched by adding 45 μl of Tris (1 M, pH 8) and Triton X-100 (20%), respectively. The cell suspension was centrifuged at 500×***g*** at 4°C for 5 min to remove cell debris. A total of 50 μl of the supernatant was reserved as the input control. For 650 μl of the supernatant, 5 μl of antibody was added and incubated for 1 h at 4°C. Next, 50 μl of a 20% protein A-sepharose CL-4B suspension (Cytiva, Uppsala, Sweden) was added, and incubation was continued for 1 h at 4°C. The beads were then washed three times at 150×***g*** for 30 s with PBS. The proteins were eluted from the beads by heating for 5 min at 95°C with sample buffer. For western blot analysis, 0.06 OD_600_ (corresponding to 2.4 × 10^6^ cells) was loaded as input, 0.6 OD_600_ (2.4 × 10^7^ cells; 10-times input) for the primary IP, and 3 OD_600_ (1.2 × 10^8^ cells; 50-times input) for the co-immunoprecipitation (co-IP). The resulting western blots were analyzed and quantified using GelAnalyzer 23.1 (www.gelanalyzer.com). The co-IP signals were normalized to the signal of the primary IP.

### Fluorescence microscopy

Yeast cells were grown overnight in SD/CAS medium at 30°C to the exponential phase. Superfolder green-fluorescent-protein-tagged (sfGFP-) Mos10 was examined with an AXIO Imager M1 equipped with a Colibri 7 light source (Zeiss, Oberkochen, Germany). The images were taken with an Axiocam 506 mono digital camera. The cells were fixed to concanavalin A-coated glass slides before imaging.

### Mutagenesis

Mutations in the *MOS10* gene were generated by error-prone PCR. A 50 μl PCR reaction was set up with 4 U Taq polymerase (Taq NEB M0273; New England Biolabs, Frankfurt, Germany), NEB standard buffer containing 1.5 mM MgCl_2_, 0.5 mM dNTPs, 500 nM primers, 0.5 mM MnCl_2_, and 2 ng pRK2061 template DNA. To amplify the *MOS10* gene, we performed 35 cycles. A mixture of the amplified PCR fragment and the vector YCplac33 cut with *EcoRI*/*HindIII* was transformed into the MOS10 deletion strain RKY3565 carrying the *CUP1p-STE6-HIS3* cassette. The *MOS10* gene was incorporated into the cut vector by homologous recombination within the yeast cell. Transformants were plated onto SD-plates containing the required auxotrophic markers (leucine, lysine, and tryptophan) and 0.5 mM Cu_2_SO_4_. His^+^ and Ura^+^ transformants were selected on these plates.

### AlphaFold modeling

Protein structure predictions were performed with AlphaFold Server, a web service based on AlphaFold3 developed by Google DeepMind that offers customized biomolecular structure prediction (https://alphafoldserver.com/) [20]. The structure predictions reported in the present study were generated on 2025-07-01. The UniProt IDs of the sequences used are P39929⋅SNF7_YEAST (for Snf7) and Q03390⋅VPS60_YEAST (for Mos10/Vps60). To evaluate the confidence of the AlphaFold3 predictions, the pLDDT and pTM scores were used. The pLDDT score indicates the per-atom accuracy of the structure prediction. AlphaFold is highly confident about the prediction at values above 90, still confident at values between 70 and 90, but has low to very low confidence at values below 70 or 50 (for details, see [20]). In the images of the structures, the pLDDT score is indicated by the coloring of the protein backbone. Dark and light blue correspond to very high or high confidence and yellow and orange to low or very low confidence. The pTM score measures the accuracy of the prediction for the entire structure. A pTM score above 0.5 means that the prediction might be similar to the true structure [20].

## Results

### Influence of mutations in endosomal protein sorting on the composition of ESCRT-III

Before we start discussing the data, we have to define what we mean by ‘ESCRT-III’ in the present article. We use the term in an operational sense as the proteins that can be co-immunoprecipitated with antibodies against ESCRT-III subunits. This leaves open the question of whether we are dealing with a single complex or with different complexes sharing common subunits. In the following, the terms ‘Mos10 complex’ or ‘Mos10-containing complex’ are used interchangeably. By this we mean ‘ESCRT-III’ immunoprecipitates containing the Mos10 protein.

Evidence has been presented that Mos10 forms an ESCRT-III complex distinct from the Snf7-containing complex [13,16]. Available data are compatible with the view that the two complexes act sequentially in endosomal protein sorting. In fluorescence microscopy images, Mos10-sfGFP displays a staining pattern that is distinct from the staining pattern of the other canonical ESCRT-III proteins. While these sfGFP-tagged proteins are localized to endosomal dots [16], Mos10-sfGFP accumulates at the vacuolar membrane [14,16], together with endosomal cargo proteins destined for degradation in the vacuole [16]. This staining most likely results from a block in Mos10 function, since, like other ESCRT-III proteins [21], C-terminally tagged Mos10 is partially non-functional. Thus, fusion of late endosomes with the vacuole seems to be blocked when Mos10 is tagged at its C-terminus, presumably due to lack of disassembly of the Mos10 complex.

We now wanted to dissect the series of events during late endosomal protein sorting by looking at the composition of ESCRT-III in different mutants of the pathway. We were especially interested in finding a condition where ESCRT-III is trapped in a form containing only Mos10 and no Snf7. To this end, ESCRT-III was immunoprecipitated from cell extracts with antibodies directed against the ESCRT-III proteins Did2, Vps2, and Vps24. The immunoprecipitates were then examined for the presence of the ESCRT-III proteins Did2, Mos10, Snf7, Vps2, and Vps24 by western blotting. All experiments were performed in triplicate. As an example, the results for the wild-type strain are shown in Supplementary Figure S1. The co-IP efficiencies for Mos10, Snf7, Vps2, and Vps24 were very similar, indicating that these proteins are present in roughly equimolar amounts in the precipitated complexes. As reported previously [12], Did2 was present in 2- to 3-fold higher amounts than the other ESCRT-III proteins. Similar results were obtained with the three different antibodies. Therefore, the data for the three antibodies were combined into a single value (Figure 1). The individual co-IP efficiencies are depicted in Supplementary Figures S2 and S3.

**Figure 1:**
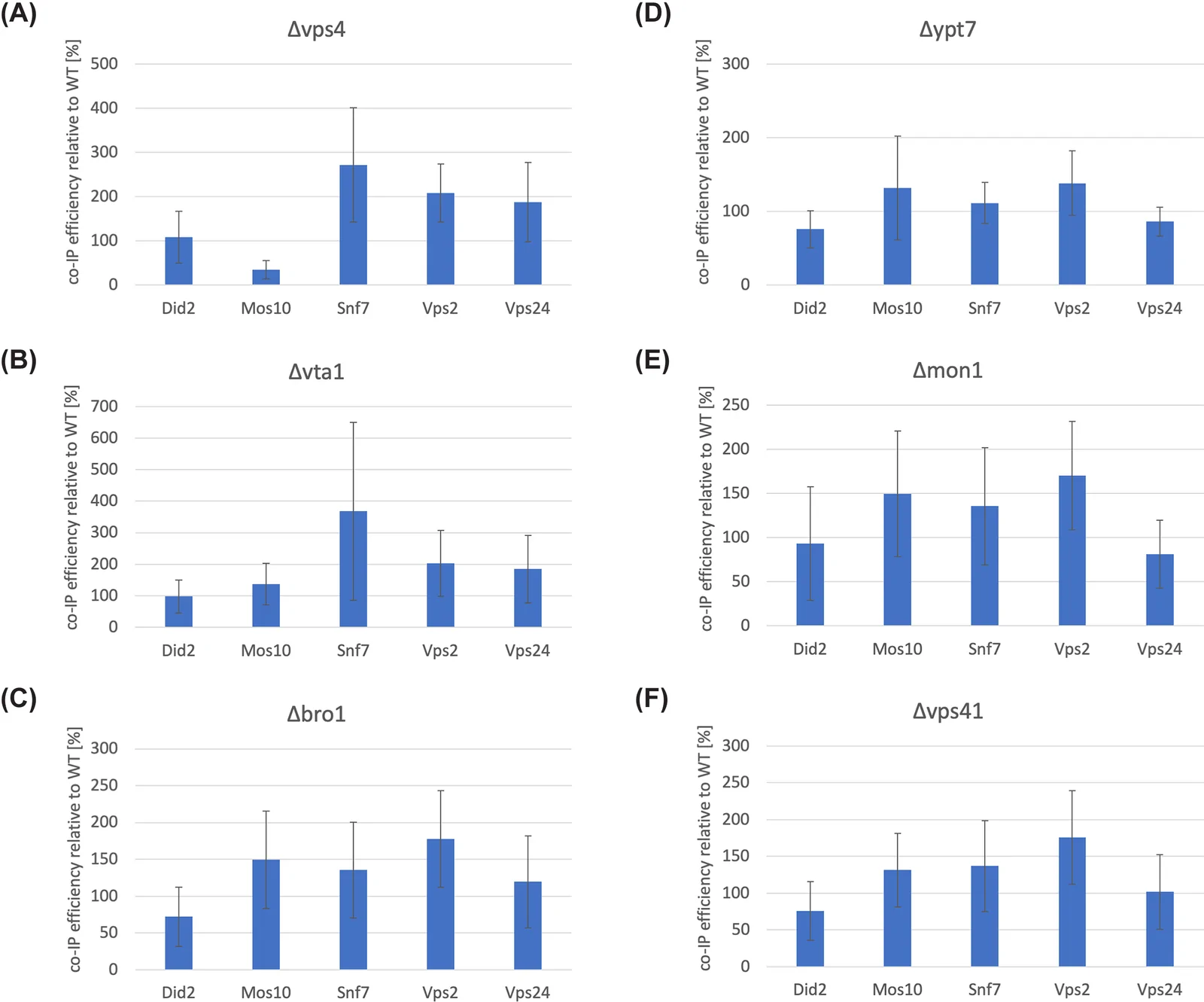
Composition of ESCRT-III in different mutant strains ESCRT-III proteins were immunoprecipitated from yeast cell extracts with anti-Did2, anti-Vps2, and anti-Vps24 antibodies. The immunoprecipitates were examined for co-IP of ESCRT-III proteins by western blotting. Relative co-IP efficiencies normalized to wild type (expressed in percent of wild type, with standard deviations) are presented. The results obtained with the three different antibodies were combined into a single value. The individual co-IP efficiencies are depicted in Supplementary Figures S2 and S3. (**A**) RKY1511 *∆vps4*, (**B**) RKY2755 *∆vta1*, (**C**) RKY1922 *∆bro1*, (**D**) RKY3504 *∆ypt7*, (**E**) RKY3642 *∆mon1*, and (**F**) RKY3502 *∆vps41*.

First, the composition of ESCRT-III was examined in a *∆vps4* mutant. The AAA+ ATPase Vps4 is an integral part of the ESCRT system. It has been implicated in the disassembly of ESCRT-III [15]. In the *∆vps4* mutant, ESCRT-III proteins as well as endocytic cargo proteins accumulate in a large dot-like structure at the vacuole, the so-called ‘class E compartment,’ in fluorescence microscopy images [22]. In line with previous reports [23], the amount of Snf7, Vps2, and Vps24 was about two-fold higher in the precipitated complexes in the *∆vps4* mutant compared with wild type (Figure 1). In contrast with these ESCRT-III proteins, the association of Did2 with ESCRT-III complexes was unaffected by the loss of Vps4 activity. An interesting result was obtained for Mos10. Its amount in the precipitated complexes was markedly reduced by the *VPS4* deletion. This suggests that Vps4 activity is necessary for the recovery of Mos10 complexes. This is in line with the notion that the Snf7-containing complex has to be disassembled before the Mos10-containing complex can be formed.

Next, the *∆vta1* mutant was examined. Vta1 is a crucial co-factor of Vps4 that stabilizes the oligomeric form of Vps4 and stimulates its ATPase activity [24]. Also, evidence has been presented that Vta1 binds to Did2 and Mos10 through its two N-terminal MIT domains [24]. Based on these findings, one would expect that the *VTA1* deletion had a similar phenotype as the *VPS4* deletion. And indeed, the co-IP pattern was very similar to the pattern of the *∆vps4* mutant, with the notable exception of Mos10, whose association with ESCRT-III complexes was not altered in the *∆vta1* mutant compared with wild type (Figure 1). Thus, Vta1 is not essential for the incorporation of Mos10 into ESCRT-III complexes. But also, similar to Did2, no accumulation of the Mos10-containing complex was observed in the *∆vta1* mutant.

Another protein, which has been identified as an interaction partner for Mos10, is the ALIX homologue Bro1. Bro1 appears to be a central hub in the sorting of proteins into intraluminal vesicles at late endosomes. It binds to Mos10, Snf7, Vps20, and ubiquitin [25–27]. In the *∆bro1* mutant, all tested ESCRT-III proteins, except Did2, showed a moderate increase in the amount of precipitated protein compared with wild type (Figure 1). Did2 did not seem to be affected or was even slightly reduced compared with wild type. Thus, Bro1 is not required for the recruitment of Mos10 to ESCRT-III complexes, and in this mutant, Mos10 behaves like the other canonical ESCRT-III proteins.

The finding that Mos10-sfGFP and endosomal cargo proteins accumulate at the vacuolar membrane [14,16] suggested that Mos10 could play a role in the fusion of endosomes with the vacuole. If endosome fusion with the vacuole is prevented, one would expect to see an accumulation of Mos10-containing ESCRT-III complexes. To test this idea, the composition of ESCRT-III was examined in mutants affected in the tethering and fusion of endosomes to the vacuole. The mutants tested were *∆ypt7, ∆mon1*, and *∆vps41*. Ypt7 is the yeast Rab7 homologue, which orchestrates tethering and fusion of endosomes to the vacuole [28]. Mon1 is a subunit of the Mon1-Ccz1 guanine nucleotide exchange factor for Ypt7 [29], and thus essential for Ypt7 activity, and Vps41 is part of the ‘homotypic fusion and vacuole protein sorting’ complex required for endosome tethering to the vacuole [30]. All three mutants showed a similar ESCRT-III composition profile with a modest increase in the amount of Mos10, Snf7, and Vps2 and unchanged levels of Did2 and Vps24 (Figure 1). Thus, our prediction that Mos10 accumulates when endosome tethering and fusion are blocked could not be confirmed.

### Intracellular distribution of Mos10-sfGFP in endosomal protein sorting mutants

Next, we were interested to see how the tested mutations affect the intracellular localization of Mos10-sfGFP (Figure 2). As already mentioned above, there is a caveat to this experiment. The Mos10-sfGFP fusion is partially defective in MVB sorting [16]. But the fusion protein maintains part of its functionality, since the phenotype of the sfGFP fusion is clearly different from a complete knock-out. With this precaution in mind, the Mos10-sfGFP fusion can still be a useful marker. The co-localization of Mos10-sfGFP with other organelle markers had been examined previously [16].

**Figure 2:**
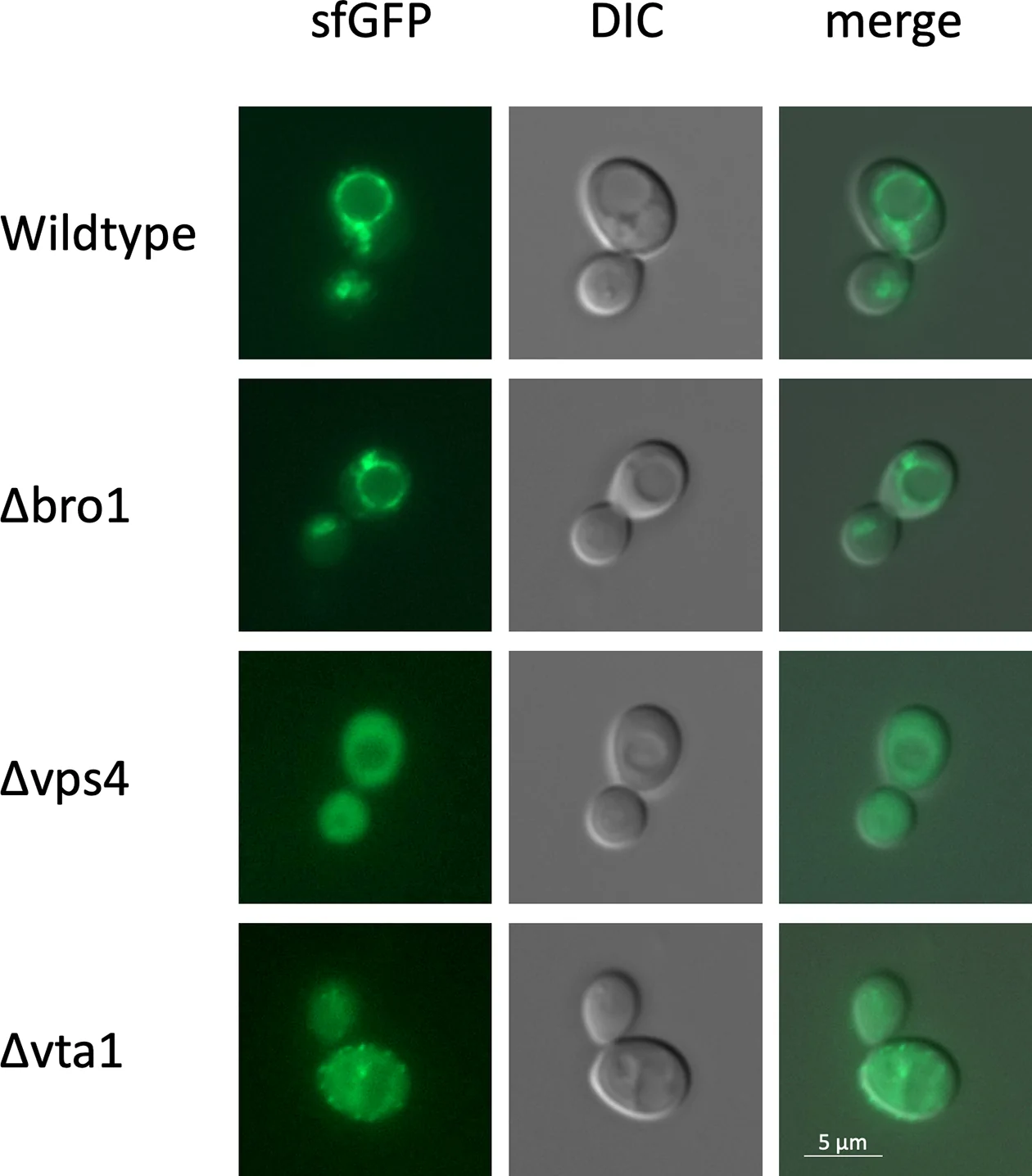
Intracellular localization of Mos10-sfGFP The localization of Mos10-sfGFP was examined by fluorescence microscopy in different mutant strains. From top to bottom: RKY3216 *wild type*, RKY3539 *∆bro1*, RKY3496 *∆vps4*, and RKY3495 *∆vta1*. Panels from left to right: sfGFP staining, DIC image, and merged image.

A pronounced effect on the localization of Mos10-sfGFP was seen in the *∆vps4* mutant. The Mos10-sfGFP staining was completely cytosolic. This finding is perfectly in line with the marked drop in the amount of Mos10 in the ESCRT-III complexes observed by co-immunoprecipitation. Thus, it appears that Mos10 is not associated with membranes in the *∆vps4* mutant. An interesting shift in the intracellular distribution of Mos10-sfGFP was seen with the *∆vta1* mutant. The protein no longer accumulated around the vacuole but was instead dispersed into a large number of tiny, mostly cortical dots, similar to the phenotype of the *∆vps68* mutation, as described previously [16]. The distribution in the *∆bro1* mutant was indistinguishable from wild type. The endosome tethering and fusion mutants (*∆mon1, ∆vps41*, and *∆ypt7*) are not included here because their vacuole fragmentation phenotype makes it difficult to interpret the staining pattern.

### Isolation of Mos10 mutants

To further dissect the interactions within the Mos10-containing ESCRT-III complex, we set out to isolate *MOS10* mutants with disrupted Mos10 function. We employed a screen previously used to isolate *SNF7* mutants [31]. The short-lived endocytic cargo protein Ste6, fused to His3, was expressed from the *CUP1* promoter. The *HIS3* gene codes for imidazole-glycerol-phosphate dehydratase, an enzyme involved in histidine biosynthesis. The Ste6-His3 fusion confers histidine prototrophy to a His3^−^ strain when expressed in sufficient amounts. But, since the Ste6-His3 fusion is degraded quickly under normal conditions [32], the level of Ste6-His3 is too low to promote growth on media lacking histidine. However, when the degradation of Ste6-His3 is blocked, as in a *MOS10* mutant, His3 activity accumulates to levels sufficient for growth on media lacking histidine. Expression of Ste6-His3 can be turned on by the addition of copper ions to the medium. *MOS10* mutations were generated by mutagenic PCR. The mutagenized *MOS10* copies were transformed into the reporter strain, and colonies were selected for growth on histidine plates.

Many of the selected mutants contained premature stop codons in the *MOS10* ORF or lacked the normal stop codon, thus expressing C-terminal extended Mos10 variants. Missense mutations were selected for further study. Most of the mutants contained multiple mutations, but all the missense mutations localized exclusively to α-helical segments or the N-terminus of Mos10. No mutations were detected in the C-terminal part of Mos10 (Figure 3). Mutant 29 (M29) was the only mutant with a single mutation localized to α-helix 1 (L49P). M34 had three mutations exclusively in α-helix 4 (E139V, L150P, and M156V). All the other mutations were spread over several Mos10 motifs. At this point it is not clear which of the mutations are actually causing the phenotype.

**Figure 3:**
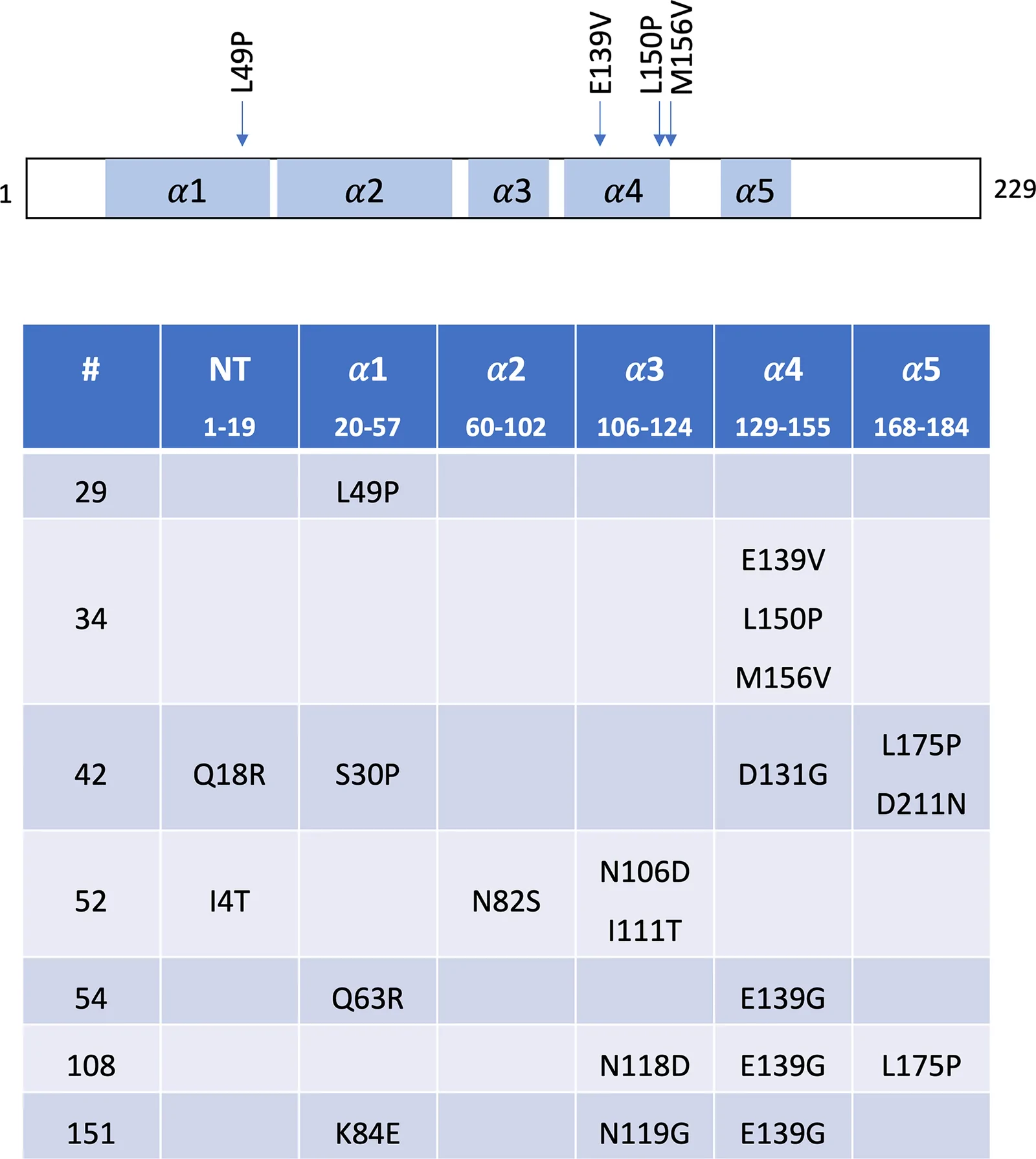
Mos10 mutations obtained by error-prone PCR mutagenesis On top of the diagram, the secondary structure of Mos10 with its five alpha-helices is depicted. The positions of the amino acids mutated in M29 and M34 are indicated. Below: list of the mutations detected in the different mutants assigned to the Mos10 secondary motifs.

### Intracellular distribution of mutant Mos10 variants

The intracellular distribution of seven mutant Mos10-sfGFP fusion proteins was examined by fluorescence microscopy (Figure 4). Several different patterns were observed. M54 with one mutation each in α1 and α4 and M151 with mutations in α1, α3, and α4 looked like wild type. Most informative were M29 and M34, because in these mutants only a single sequence motif of Mos10 is affected. Instead of the staining surrounding the vacuole, M29 displayed a handful of endosomal dots. This staining resembled the staining previously observed with other ESCRT-III proteins like Did2, Snf7, Vps2, and Vps24 [16]. Thus, a mutation in α1 appears to block the progression to the Mos10-containing complex, which finally ends up at the vacuole. A staining similar to M29 was observed with M52, which carries mutations in the N-terminus, in α2 and α3. An interesting phenotype was observed with M34; this protein was completely cytosolic. Since its mutations are exclusively localized to α4, this mutant highlights the central importance of α4 for the interaction with the other ESCRT-III proteins. To see which of the three mutations in M34 is responsible for the observed phenotype, variants with single mutations were generated. As can be seen in Supplementary Figure S4, the M34 phenotype is recapitulated by the L150P mutation. The E139V and M156V mutations gave rise to a wild-type pattern. Cytosolic staining was also observed for M108 with mutations in α3, α4, and α5. Yet another staining pattern was observed with M42 with mutations in the N-terminus, α1, α4, and α5. This mutant protein was mostly cytosolic, but with additional faint dots. This looks like a staining pattern intermediate between M29 and M34.

**Figure 4:**
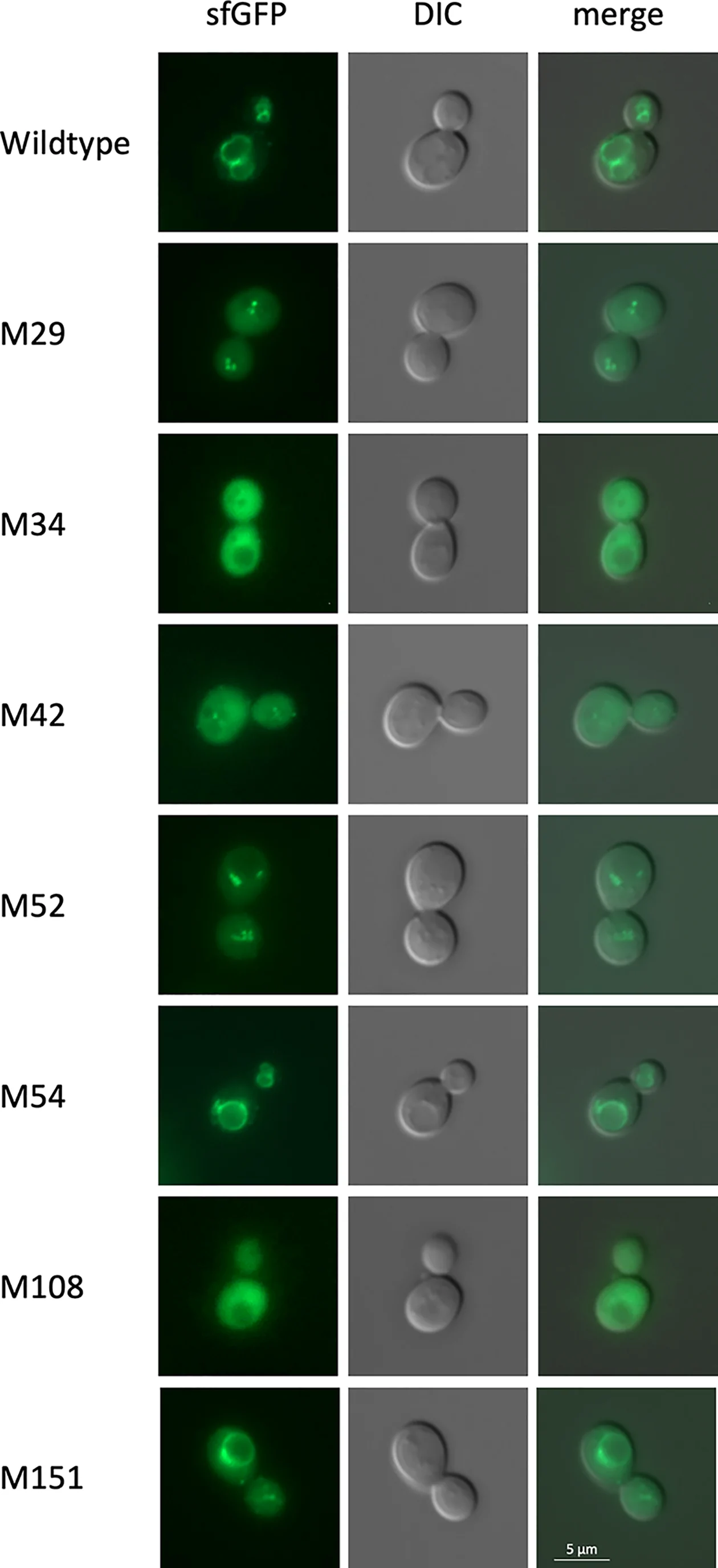
Intracellular localization of mutant Mos10-sfGFP proteins The mutant *MOS10* genes were cloned into plasmid pRK1691 expressing Mos10-sfGFP fusions. The plasmids were transformed into the *∆mos10* strain RKY2892, and then the transformants were examined by fluorescence microscopy. From top to bottom: pRK1957 wild type Mos10, pRK2126 M26, pRK2127 M34, pRK2128 M42, pRK2129 M52, pRK2130 M54, pRK2131 M108, pRK2132 M151. Panels from left to right: sfGFP staining, DIC image, and merged image.

The fluorescence microscopy results suggest that α4 is crucial for the interaction with other ESCRT-III proteins. To test this directly, a two-hybrid experiment was performed. The Gal4 activation domain (Gal-AD) was fused to Mos10, and the other ESCRT-III proteins were fused to the Gal4 DNA-binding domain (Gal-BD), and the constructs were transformed into the two-hybrid reporter strain [33]. As can be seen in Figure 5, all Gal-BD-ESCRT-III fusions tested led to an activation of the *HIS3* reporter gene in combination with Gal-AD-Mos10. Thus, Mos10 appears to be able to bind to all ESCRT-III proteins tested, at least to a certain degree. But, most interestingly, the interactions were completely abrogated by the M34 mutations. This supports the notion that the α4 helix of Mos10 is central to the ESCRT-III interactions. In this respect, Mos10 resembles Snf7. For Snf7, it has been shown that its α4 helix is crucial for the interaction with Vps24. Mutations in this helix lead to lack of recruitment of Vps24 to ESCRT-III [34].

**Figure 5:**
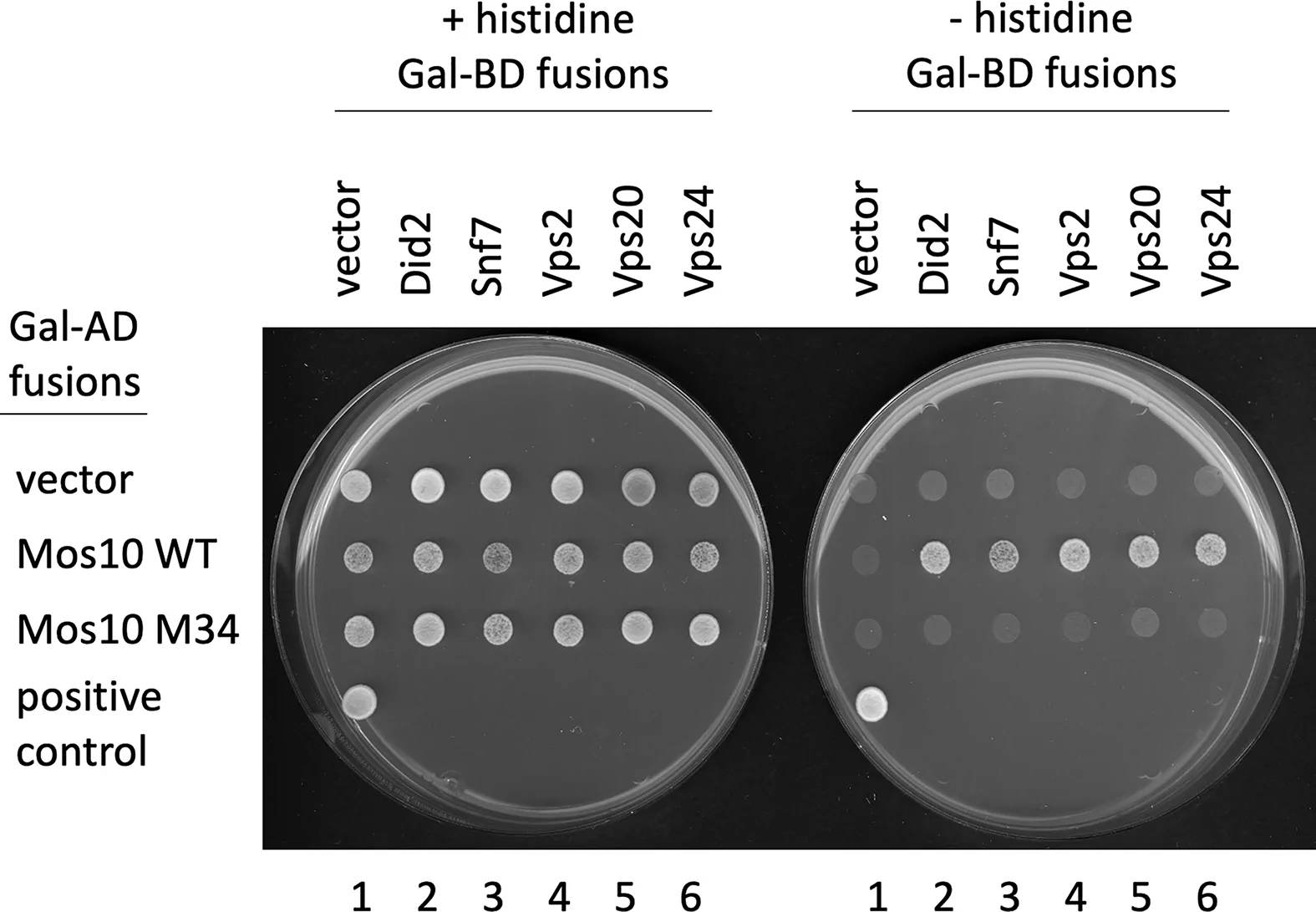
Two-hybrid analysis of Mos10 ESCRT-III interactions Wild-type *MOS10* or *mos10-M34* fused to the Gal4-AD were transformed into the two-hybrid reporter strain PJ69-4A in combination with ESCRT-III genes fused to the Gal-BD. The transformants were spotted onto agar plates containing histidine (left) or lacking histidine (right). Horizontal order of plasmids: (1) pRK329 (Gal-BD vector), (2) pRK1532 (Gal-BD-Did2), (3) pRK942 (Gal-BD-Snf7), (4) pRK1533 (Gal-BD-Vps2), (5) pRK842 (Gal-BD-Vps20), (6) pRK1534 (Gal-BD-Vps24). Vertical order of plasmids: pACT2 (Gal-AD vector), pRK1870 (Gal-AD-Mos10), pRK2187 (Gal-AD-Mos10-M34), single spot = positive control, pSE1111 (Gal-AD-Snf4), pSE1112 (Gal-BD-Snf1). The plates were incubated for 2 d at 30°C.

### Modeling with AlphaFold

AlphaFold modeling [20] was used to predict the impact of the L49P and L150P mutations on the Mos10 structure. Wild-type Mos10 was modeled by AlphaFold in its extended open conformation, similar to the open conformation described for Snf7 [35] (Supplementary Figure S5). In this conformation, the α3 helix aligns with the α2 helix, forming a long α-helical region. The α-helical regions from α1 to α4 are modeled with high to very high confidence. However, in line with X-ray crystallographic studies on other ESCRT-III proteins [35], the confidence level of the prediction is low in the N-terminal and C-terminal regions due to high structural flexibility. The pTM score for wild-type Mos10 was 0.48. The somewhat lower score is due to ‘dilution’ of the score by the flexible N- and C-terminal regions. This has to be kept in mind when judging the pTM scores of the following predictions, which are all lower than 0.5. The introduction of the L49P mutation had no significant impact on the predicted Mos10 structure (pTM = 0.47; Supplementary Figure S5).

Two molecules of Mos10 are predicted to form a crescent-shaped tail-to-tail dimer with the N-termini at the convex side of the crescent (pTM = 0.37; Supplementary Figure S6). According to this prediction, the two monomers interact with each other in an anti-parallel fashion via a long α-helical region extending from the C-terminal end of α2 to the end of a shortened α4 helix. The predicted structure of the Mos10 dimer changed slightly when the L49P mutation was introduced into the Mos10 sequence (Supplementary Figure S6). The α4 helix now had the same length as in the Mos10 monomer structure, and the N-termini of the two monomers were farther apart from each other compared with the wild type. The confidence level of the L49P dimer structure was somewhat lower compared with the wild type (pTM = 0.3). But an intriguing effect of the L49P mutation was observed when a Mos10–Snf7 dimer was modeled (Figure 6). The predicted Mos10–Snf7 dimer (pTM = 0.28) resembled the Mos10 dimer in its overall arrangement. But when the L49P mutation was introduced into Mos10, the tail-to-tail dimer was converted into a side-by-side dimer (pTM = 0.3), where the two α1/α2 hairpins align with each other, which is exactly the reported arrangement of monomers in the 3D structure of an Snf7 filament [35]. Thus, if Mos10 and Snf7 do indeed interact, the L49P mutation could alter the way of their interaction dramatically.

**Figure 6:**
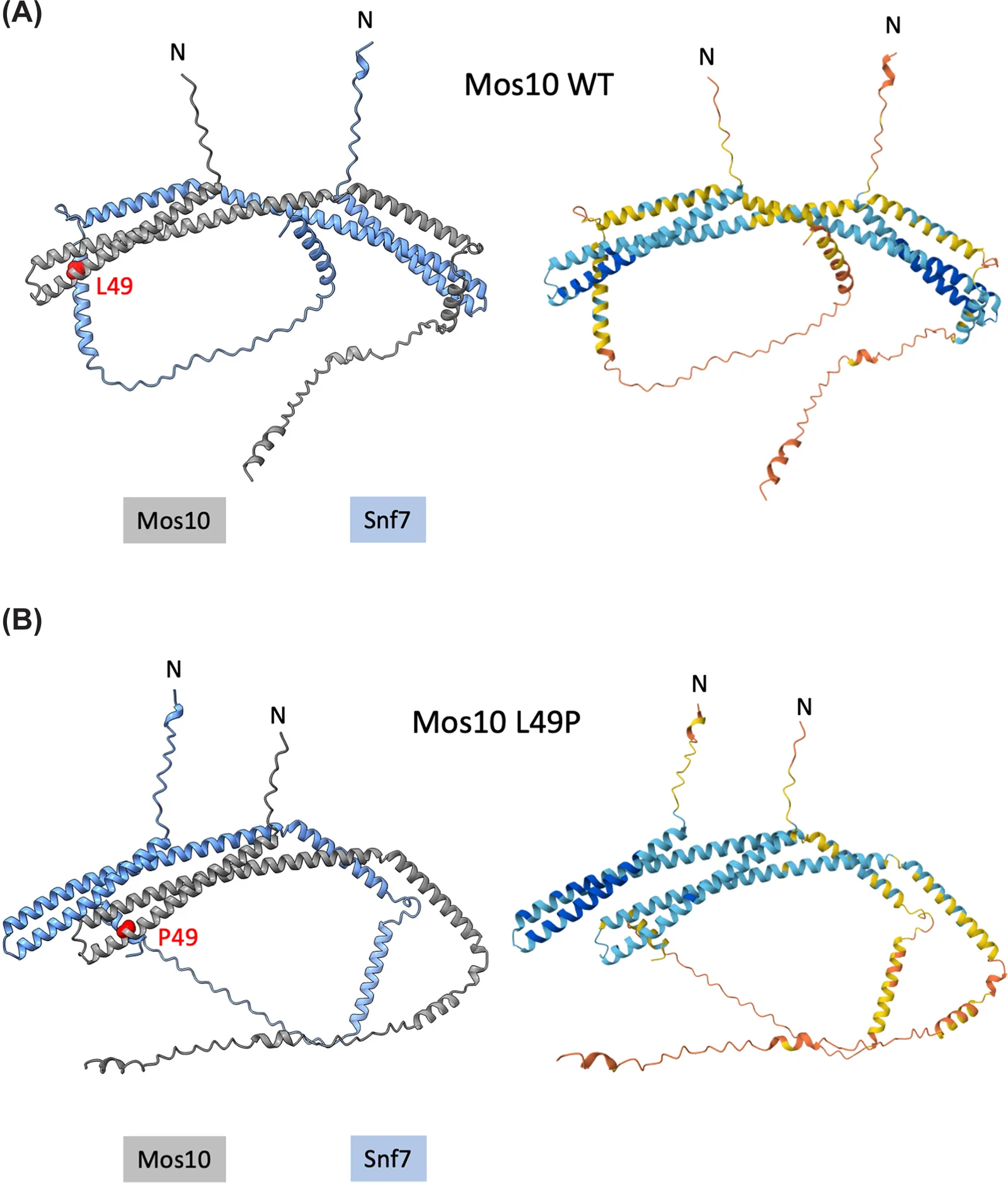
Modeling of a Mos10–Snf7 dimer by AlphaFold (https://alphafoldserver.com/) (**A**) Dimer of wild-type Mos10 and Snf7. (**B**) Dimer of Mos10-L49P and Snf7. Left panels: Mos10 = gray, Snf7 = blue. Right panels: AlphaFold pLDDT coloring scheme. The residue at position 49 is highlighted by a red sphere. The N-termini of the proteins are marked.

Next, the impact of the L150P mutation was examined. The introduction of this mutation into α4 had no noticeable effect on the predicted Mos10 structure (pTM = 0.49; Supplementary Figure S5). Although pairwise modeling of Mos10 with the other ESCRT-III members was compatible with a potential role of Mos10 α4 in the interaction with Vps2 or Vps24 (not shown), no reliable conclusions about the role of the L150P mutation on these potential interactions could be drawn due to the low confidence level of these predictions.

## Discussion

In the present study, we explored the relationship between the Snf7 and Mos10-containing ESCRT-III complex by looking at the composition of ESCRT-III in different mutants of the endosomal protein sorting pathway and by isolation of Mos10 mutants.

*In vitro* data suggested that Mos10 and Snf7 form mutually exclusive complexes with the same ESCRT-III partners (Did2, Ist1, Vps2, and Vps24) [13], but it is not clear whether this is also true *in vivo* in a yeast cell. Since both complexes appear to be very similar in their composition with respect to the other ESCRT-III binding partners, we probably pull out a mixture of these two complexes in our co-IP experiments if there are two distinct complexes. For the co-IPs, we used antibodies against the class II ESCRT-III proteins Did2, Vps2, and Vps24 shared by both complexes. Immunoprecipitations against the class I ESCRT-III proteins with anti-Mos10 and anti-Snf7 antibodies proved difficult to interpret due to substantial cross-reactivity between the two antisera [12].

If separate Mos10 and Snf7 complexes exist in the cell, it should be possible to create situations where only one or the other complex is present. We were successful in identifying such a situation by looking at the composition of ESCRT-III in the *∆vps4* mutant. The AAA+ ATPase Vps4 is thought to be required for the disassembly of ESCRT-III [15]. Previous experiments showed that the canonical ESCRT-III proteins Snf7, Vps2, and Vps24 accumulate at endosomal membranes in this mutant [23]. But, for Mos10, we observed the opposite effect; it was strongly depleted from the precipitated ESCRT-III complexes, while the amount of the canonical ESCRT-III proteins was increased. In line with our findings, it was noted previously that the size of Snf7 polymers was larger in a *∆vps4* mutant compared with wild type, while Mos10 was present in its unpolymerized form [13]. The biochemical experiments are nicely complemented by our fluorescence microscopy experiments, which show that Mos10-sfGFP is no longer associated with visible membrane structures but is instead completely cytosolic in *∆vps4* mutant cells. Our data thus demonstrate that Vps4 activity is essential for the recovery of Mos10-containing ESCRT-III complexes. The most straightforward interpretation of these findings is that Vps4 activity is required for the formation of Mos10-containing complexes. But alternative explanations are also possible: Loss of Vps4 activity could alter membrane recruitment of Mos10, accelerate the turnover of Mos10-containing complexes, change the epitope accessibility, or alter the membrane extractability of the complexes.

The strict dependence on Snf7 [12] suggests that Mos10-containing complexes form after the Snf7-containing complexes. But it is not clear whether mixed complexes are formed, containing Mos10 and Snf7, or whether there is a switch from an Snf7-containing complex to a Mos10-containing complex, or whether the two complexes exist side-by-side, as the *in vitro* data would suggest [13]. If there is a sequential progression from an Snf7-containing complex to a Mos10-containing complex, it should be possible to accumulate the Mos10 complex by blocking the endosomal pathway at the very last step, which is fusion of the endosome with the vacuole. But, in mutants blocked at this step (*∆mon1, ∆vps41*, and *∆ypt7*), Mos10 complexes did not accumulate at the expense of the Snf7 complexes. Instead, we even saw a slight increase in the amount of Snf7 in the ESCRT-III complexes isolated under these conditions. C-terminal tagging of Mos10 with sfGFP also leads to a block in endosome fusion with the vacuole [16]. But Snf7 was still present in the complexes isolated from the Mos10-sfGFP strain, and even in elevated amounts (Supplementary Figure S7). Thus, the fusion mutants did not provide evidence for a simple terminal accumulation of Mos10 complexes, arguing against a straightforward linear maturation model. This does not exclude more transient or spatially restricted transitions. In fact, quantitative fluorescence lattice light-sheet microscopy experiments suggested that there is a continuous, stochastic exchange of Vps4 and ESCRT-III components at the endosomal membrane during ILV formation, rather than a steady growth of fixed assemblies [21].

Taking all the available information into account, it seems possible that at some stage during ILV formation mixed complexes containing Snf7 and Mos10 are being formed. At present this is merely a working hypothesis, which needs further experimental support. The incorporation of Mos10 into the complexes could promote disassembly of ESCRT-III. A role for Mos10 in the disassembly of ESCRT-III has been suggested previously. The so-called ‘ESCRT-III-associated proteins’ Did2 and Mos10 were initially viewed as factors regulating the ATPase activity of Vps4 [9,10]. In line with a proposed function in disassembly, we also noted that canonical ESCRT-III proteins like Snf7 accumulate on membranes in a *∆mos10* mutant [16]. Also, the Mos10 complexes appear to be short-lived. Under steady-state conditions, only small amounts of Mos10 are associated with membranes, much less than in the case of the other ESCRT-III proteins (our unpublished observations). This argues rather against the side-by-side existence of stable Snf7 and Mos10 complexes.

How could Mos10 assist in the disassembly of ESCRT-III? Several models have been put forward to explain the role of Vps4 in ESCRT-III-dependent membrane remodeling, but a consensus has not been reached yet [36]. Our structural modeling with AlphaFold may offer some hint on a possible mechanism. AlphaFold predicts the formation of a tail-to-tail dimer between Snf7 and Mos10. The formation of such a dimer could disrupt the Snf7 filament by extracting Snf7 monomers from the filament or by replacing Snf7 monomers in the filament with Mos10 monomers. Such a filament may be intrinsically unstable, leading to spontaneous disassembly. This would be a variant of the dynamic exchange model, where Vps4 exchanges one ESCRT-III subunit for another, thus changing the properties of the filament [36].

An interesting rearrangement of the dimer is predicted by AlphaFold when the M29 mutant with the L49P mutation in α-helix 1 is modeled with Snf7. Mos10-L49P now aligns side-by-side with Snf7 just as the monomers in a homomeric Snf7 filament [35]. The Mos10-L49P mutation appears to block the normal progression or maturation of ESCRT-III complexes, since the transition to a vacuolar localization observed for wild-type Mos10-sfGFP does not occur. Our mutagenesis study also highlights the importance of the Mos10 α-helix 4 for the interaction with other ESCRT-III subunits. The L150P mutation in α-helix 4 prevented membrane association of the sfGFP fusion protein, probably by disrupting the interaction with other ESCRT-III subunits.

An interesting effect on the intracellular localization of Mos10-sfGFP was observed in the *∆vta1* mutant. Mos10-sfGFP was dispersed into a large number of small, mostly cortical dots. A similar phenotype was observed before for the *∆vps68* mutant [16]. The Vps55/Vps68 complex performs a parallel function with the yeast scramblase homologue Tms1 [18]. Thus, an altered lipid composition of the endosomal membrane could be responsible for the mislocalization of Mos10-sfGFP in the *∆vps68* mutant. No co-staining with endosomal cargo proteins like Ste6 and Cps1 was detected in the cortical dots (our unpublished observation). These dots therefore do not seem to correspond to mislocalized endosomes. We rather think that they correspond to carriers involved in retrograde transport to the plasma membrane. This would mean that loss of Vta1 leads to misincorporation of Mos10-containing ESCRT-III complexes into recycling tubules. Direct interaction with the MIT domains of Vta1 could help to retain Did2 and/or Mos10-containing ESCRT-III complexes in the limiting membrane of the late endosome.

## Supplementary Material

Supplementary Figures S1-S7 and Tables S1-S2

## Data Availability

All data are presented in the article.

## Open Access

Open access for this article was enabled by the participation of University of Hohenheim in an all-inclusive Read & Publish agreement with Portland Press and the Biochemical Society under an agreement with TIB German Alliance.

## Abbreviations

co-IP: co-immunoprecipitation
ESCRT: endosomal sorting complex required for transport
Gal-AD: Gal4 activation domain
Gal-BD: Gal4 DNA-binding domain
ILVs: intraluminal vesicles
M29: Mutant 29
MVBs: multivesicular endosomes
PBS: phosphate-buffered saline
sfGFP-: superfolder green-fluorescent-protein-tagged
