# Supplementary material for: Characterization of the Mos10 (Vps60) ESCRT-III complex
Source: Biosci Rep. 2026 Oct 5;46(10):BSR20260272. doi: 10.1042/BSR20260272 (PMC13643019; doi:10.1042/BSR20260272)
Supplement: Supplementary file 1 [file BSR-2026-0272-T_supp.pdf]

Tab S1: Yeast strains

Strain	Genotype	
JD52	*MATa ura3-52 his3-Δ200 leu2-3,112 trp1-Δ63 lys2-801 ssd1-d2*	J. Dohmen, Köln
PJ69-4A	*ade2 trp1-901 leu2-3,112 ura3-52 his3-Δ200 gal4Δ gal80Δ LYS2::GAL1-HIS3 GAL2-ADE2 met2::GAL7-lacZ*	James et al. 1996
RKY1511	*Δvps4::HIS3*	this study
RKY1922	*Δbro1::kan*	this study
RKY2755	*Δvta1::HIS3*	this study
RKY2892	*Δmos10*	this study
RKY3216	*MOS10-sfGFP::kan*	this study
RKY3342	*MOS10-sfGFP::His3 Δypt7::TRP1*	this study
RKY3494	*MOS10-sfGFP::kan Δvps41::HIS3*	this study
RKY3495	*MOS10-sfGFP::kan Δvta1::HIS3*	this study
RKY3496	*MOS10-sfGFP::kan Δvps4::HIS3*	this study
RKY3502	*Δvps41::HIS3*	this study
RKY3504	*Δypt7:HIS3*	this study
RKY3565	*Δmos10 suc2::[CUP1p-STE6-HIS3 kan]*	this study
RKY3642	*Δmon1::HIS3*	this study

Tab S2: Plasmids

Plasmid	Vector	Insert
pACT2		*GAL-AD*
pRK329		*GAL-BD*
pSE1111		*GAL-AD-SNF4*
pSE1112		*GAL-BD-SNF1*
YCplac22		*CEN ARS TRP1*
YCplac33		*CEN ARS URA3*
pRK842	pRK329	*GAL-BD-VPS20*
pRK942	pRK329	*GAL-BD-SNF7*
pRK1532	pRK329	*GAL-BD-DID2*
pRK1533	pRK329	*GAL-BD-VPS2*
pRK1534	pRK329	*GAL-BD-VPS24*
pRK1870	pACT2	*GAL-AD-MOS10*
pRK2187	pACT2	*GAL-AD-MOS10-M34*
pRK1691	YCplac22	*sfGFP*
pRK1957	pRK1691	*MOS10*
pRK2061	YCplac22	*MOS10*
pRK2126	pRK1957	*MOS10-M29*
pRK2127	pRK1957	*MOS10-M34*
pRK2128	pRK1957	*MOS10-M42*
pRK2129	pRK1957	*MOS10-M52*
pRK2130	pRK1957	*MOS10-M54*
pRK2131	pRK1957	*MOS10-M108*
pRK2132	pRK1957	*MOS10-M151*
pRK2184	pRK1957	*MOS10-E139V*
pRK2185	pRK1957	*MOS10-L150P*
pRK2186	pRK1957	*MOS10-M156V*

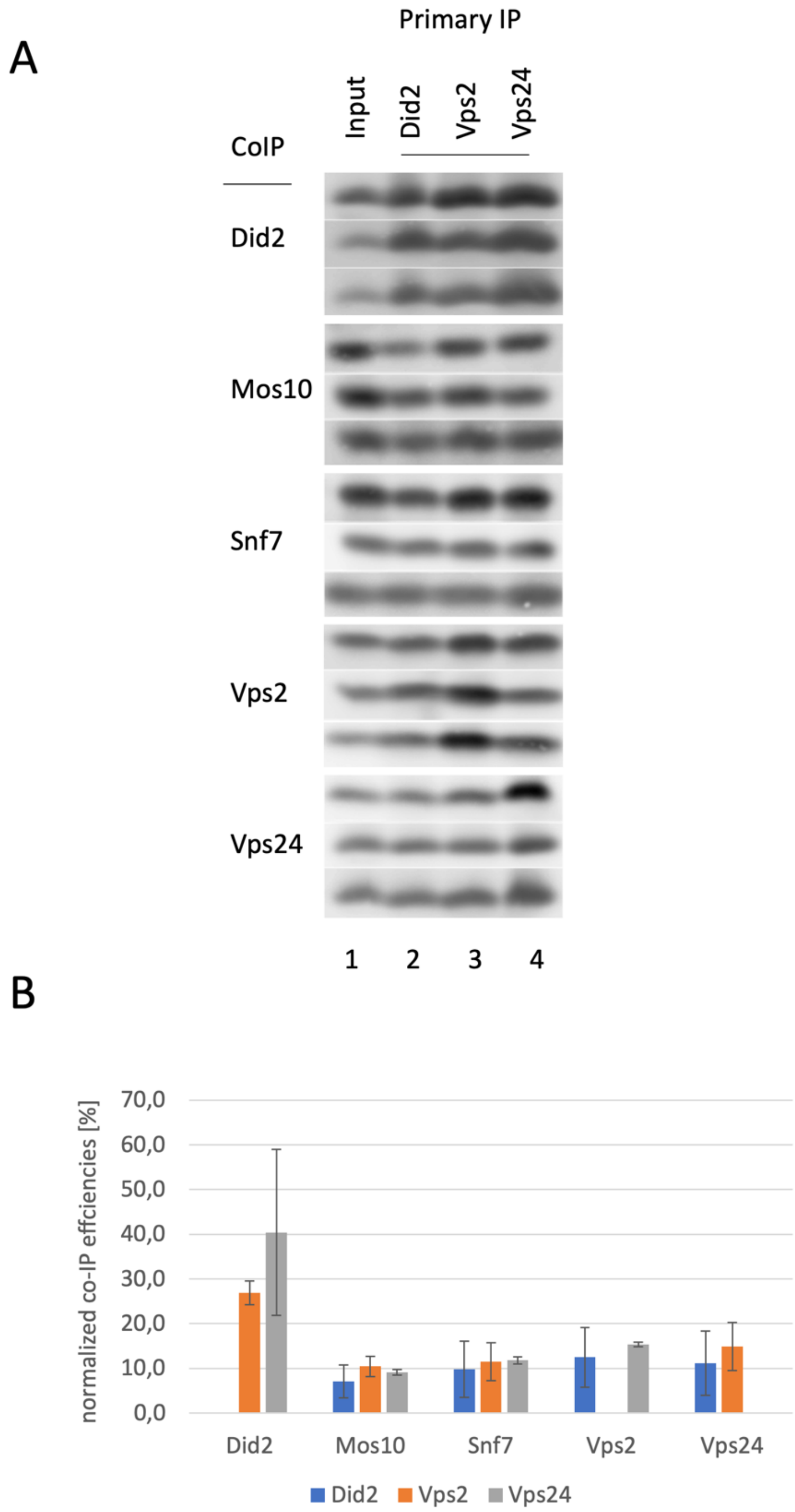

Figure S1 Co-immunoprecipitation with the wildtype strain. (A) ESCRT-III proteins were immunoprecipitated from yeast cell extracts of JD52 with (2) anti-Did2, (3) anti-Vps2 and (4) anti-Vps24 antibodies (primary IP), (1) input. The immunoprecipitates were examined for co-immunoprecipitation (co-IP) of ESCRT-III proteins by western blotting. The experiments were performed in triplicate. Western blots from top to bottom were developed with the following antibodies: anti- Did2, anti-Mos10, anti-Snf7, anti-Vps2, anti-Vps24. Input: 10 % of primary IP and 2 % of co-IP. (B) The intensities of the western blot signals were quantified. Given is the fraction of protein precipitated with respect to the input control. The primary IP efficiencies were normalized to 100 %.

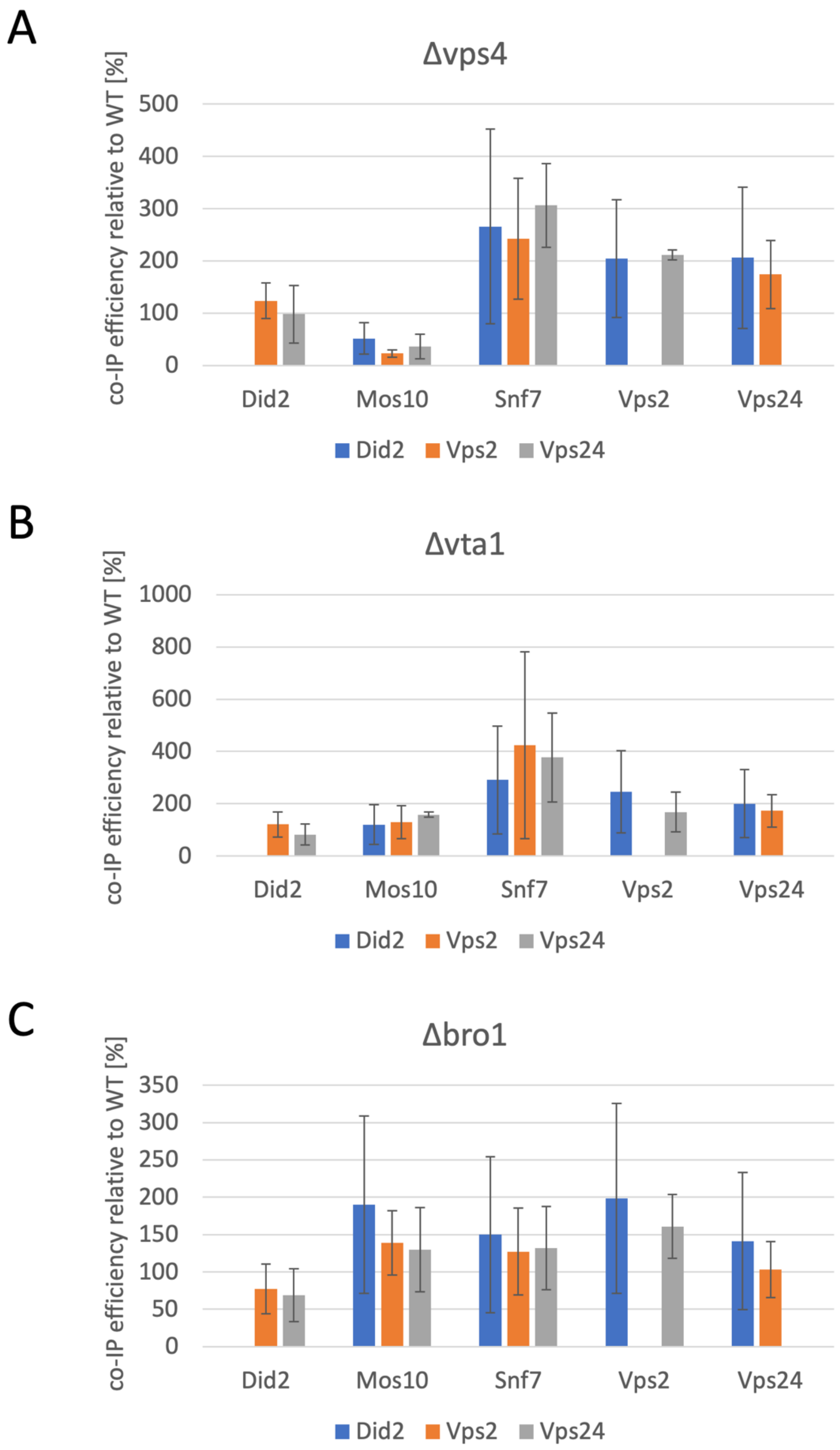

Figure S2 Individual co-IP efficiencies for the strains shown in Fig. 1. (A) RKY1511 *Δvps4*, (B) RKY2755 *Δvta1*, (C) RKY1922 *Δbro1*. Immunoprecipitations with anti-Did2, anti-Vps2 and anti-Vps24 antibodies. Relative co-IP efficiencies normalized to wildtype (expressed in percent of wildtype, with standard deviations) are presented.

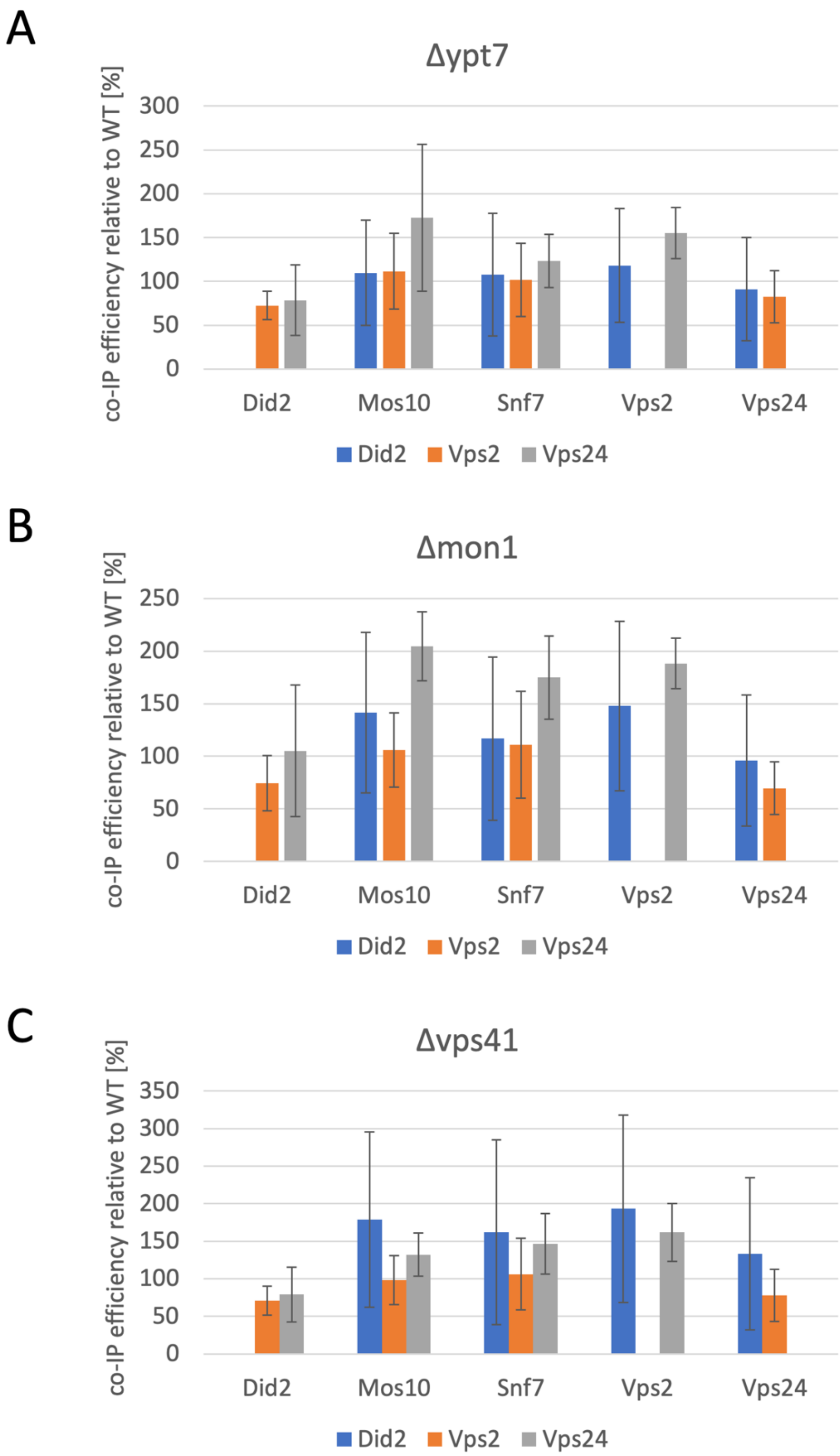

Figure S3 Individual co-IP efficiencies for the strains shown in Fig. 2. (A) RKY3504 *Δypt7*, (B) RKY3642 *Δmon1*, (C) RKY3502 *Δvps41*. Immunoprecipitations with anti-Did2, anti-Vps2 and anti-Vps24 antibodies. Relative co-IP efficiencies normalized to wildtype (expressed in percent of wildtype, with standard deviations) are presented.

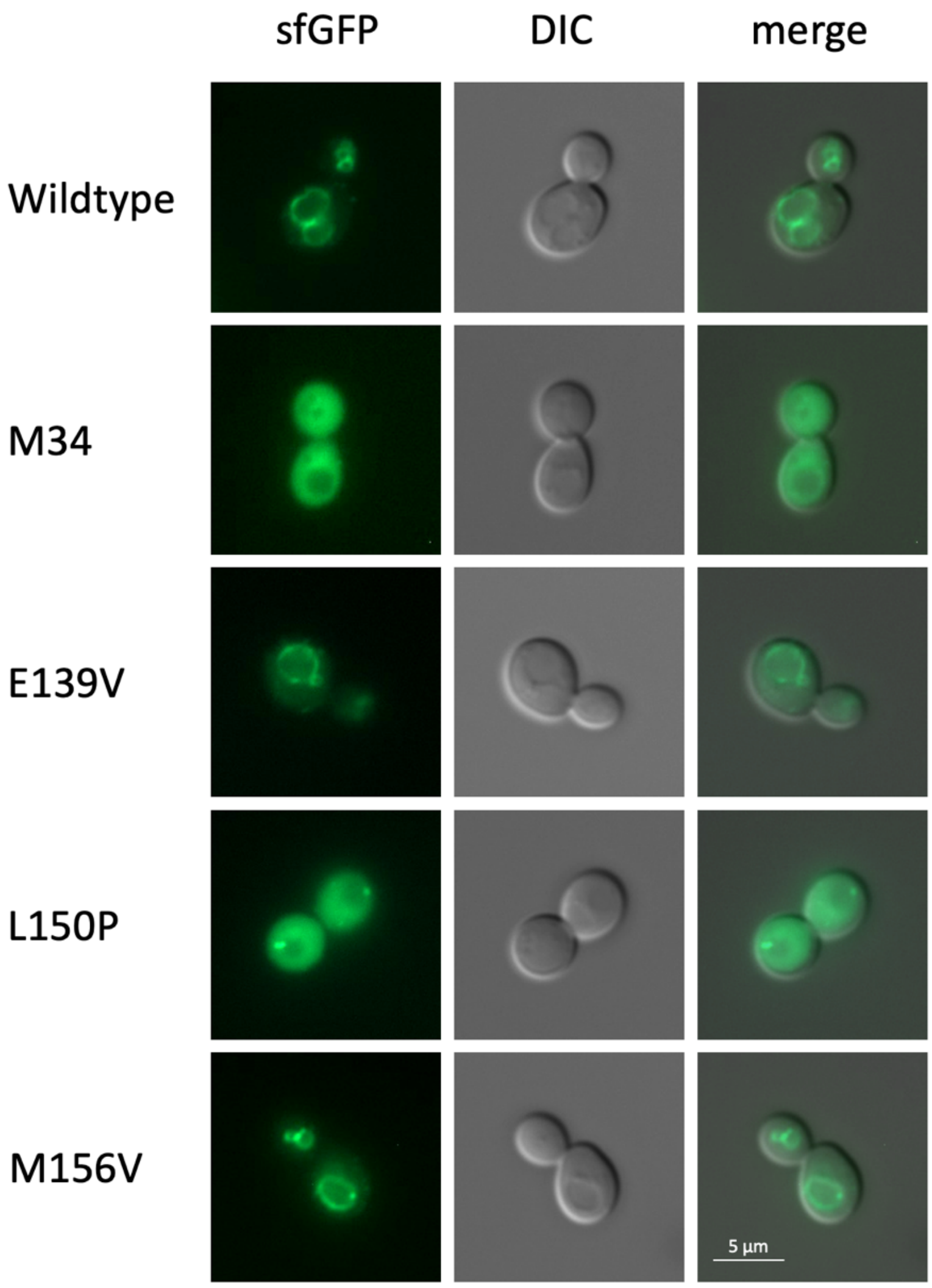

Figure S4 Intracellular localization of mutant Mos10-sfGFP proteins. The mutant *MOS10* genes were cloned into plasmid pRK1691 expressing Mos10-sfGFP fusions. The plasmids were transformed into the *Δmos10* strain RKY2892 and the transformants were examined by fluorescence microscopy. From top to bottom: pRK1957 wildtype Mos10, pRK2127 Mos10-M34, pRK2184 Mos10-E139V, pRK2185 Mos10-L150P, pRK2186 Mos10-M156V. Panels form left to right: sfGFP staining, DIC image, merged image.

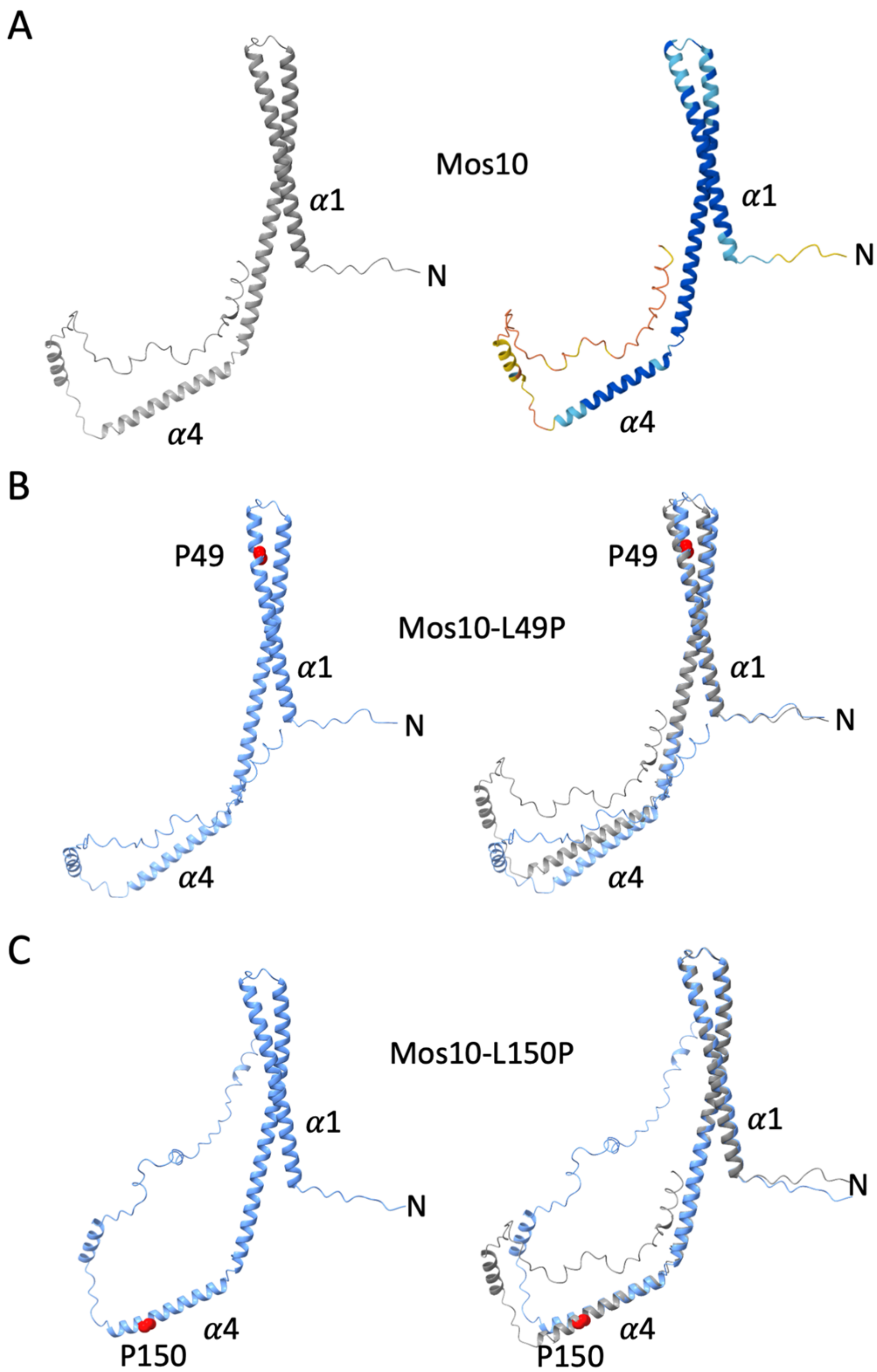

Figure S5 Modeling of wildtype Mos10, Mos10-L49P and Mos10-L150P by AlphaFold (https://alphafoldserver.com/). (A) Left panel: wildtype Mos10 (grey); right panel: wildtype Mos10 with AlphaFold pLDDT coloring scheme. (B) Left panel: Mos10-L49P (blue); right panel: superimposition of the mutant protein structure (blue) onto the wildtype protein structure (grey). (C) Left panel: Mos10-L150P (blue); right panel: superimposition of the mutant protein structure (blue) onto the wildtype protein structure (grey). The mutant residues are marked with red spheres. The N-termini and the $\alpha 1$ and $\alpha 4$ helices are indicated.

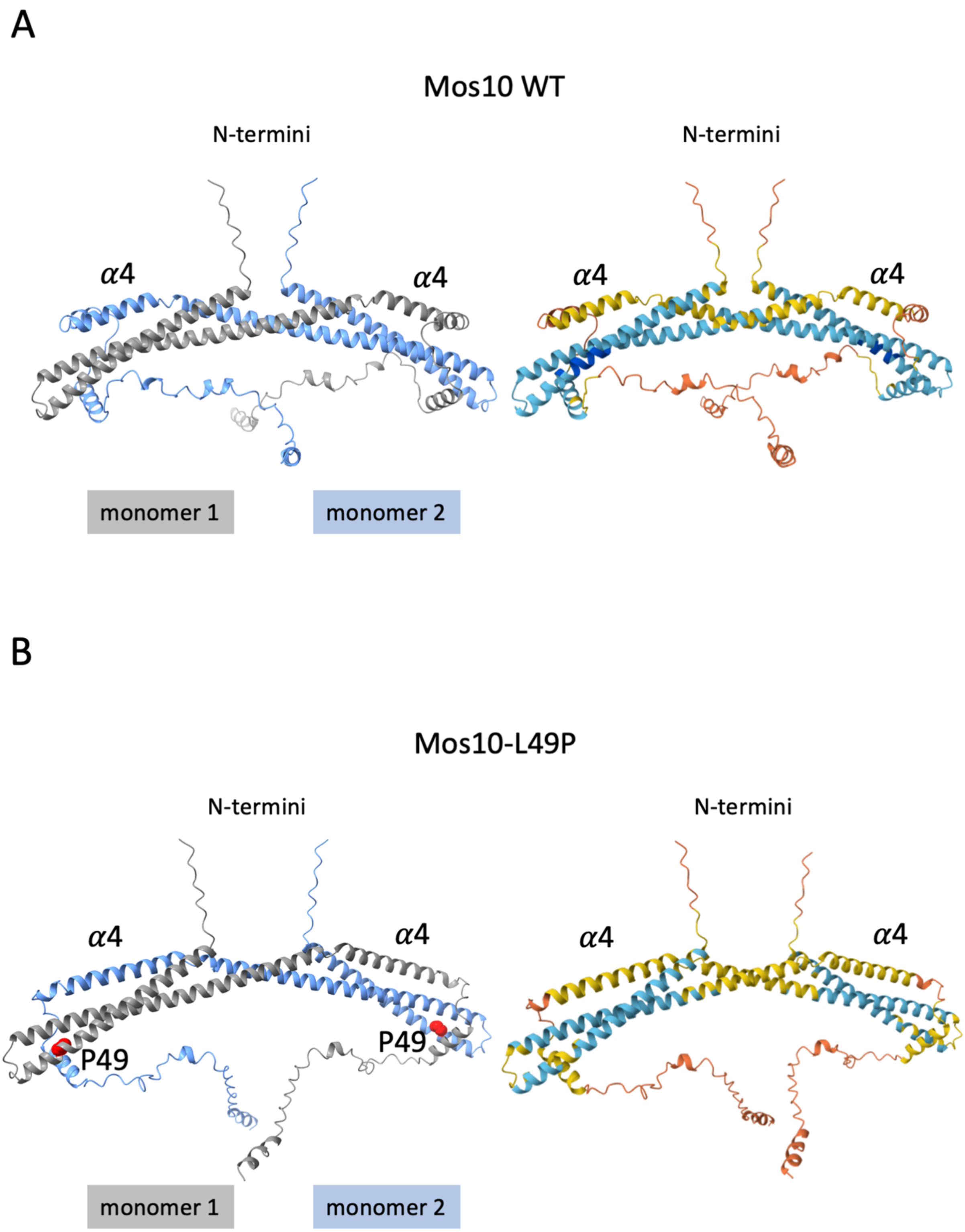

Figure S6 Modeling of wildtype Mos10 and Mos10-L49P dimers by AlphaFold (https://alphafoldserver.com/). A) Dimer of wildtype Mos10. (B) Dimer of Mos10-L49P. Left panels: For better distinction the two monomers are colored grey and blue. Right panels: AlphaFold pLDDT coloring scheme. The L49P mutation is highlighted by a red sphere. The N-termini of the proteins and the $\alpha 4$ helices are indicated.

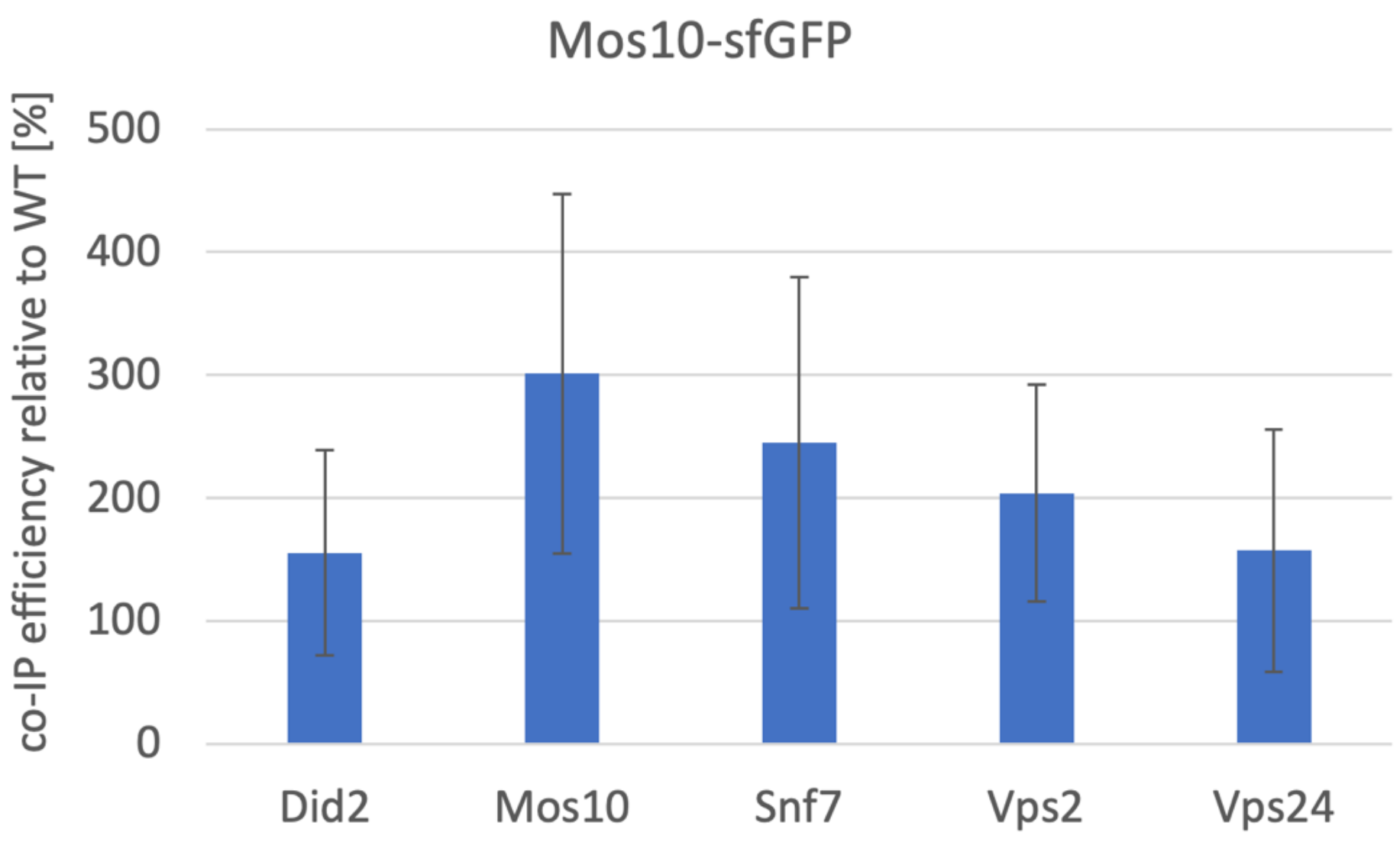

Figure S7 Composition of ESCRT-III in the strain expressing Mos10-sfGFP. ESCRT-III proteins were immunoprecipitated from yeast cell extracts of RKY3216 with anti-Did2, anti-Vps2 and anti-Vps24 antibodies. The immunoprecipitates were examined for co-immunoprecipitation (co-IP) of ESCRT-III proteins by western blotting. Relative co-IP efficiencies normalized to wildtype (expressed in percent of wildtype, with standard deviations) are presented. The results obtained with the three different antibodies were combined into a single value.
